# 2-(4-Bromo­benzene­sulfonamido)benzoic acid

**DOI:** 10.1107/S1600536809022545

**Published:** 2009-06-17

**Authors:** Muhammad Nadeem Arshad, Islam Ullah Khan, Mehmet Akkurt, Muhammad Shafiq, Ghulam Mustafa

**Affiliations:** a Materials Chemistry Laboratory, Department of Chemistry, Government College University, Lahore, Pakistan; b Department of Physics, Faculty of Arts and Sciences, Erciyes University, 38039 Kayseri, Turkey

## Abstract

In the title compound, C_13_H_10_BrNO_4_S, the dihedral angle between the benzene rings is 82.75 (15)°. An intra­molecular N—H⋯O hydrogen bond generates an *S*(6) ring motif. In the crystal structure, two mol­ecules form an *R*_2_^2^(8) centrosymmetric dimer through a pair of O—H⋯O hydrogen bonds. Intra- and inter­molecular C—H⋯O hydrogen bonds are also observed.

## Related literature

For background to sulfonamide derivatives, see: Allison *et al.* (2006 ▶); Sheppard *et al.* (2006 ▶). For related structures, see: Arshad *et al.* (2009 ▶); Shafiq *et al.* (2009 ▶); Asiri *et al.* (2009 ▶). For hydrogen-bond graph-set terminology, see: Bernstein *et al.* (1995 ▶); Etter (1990 ▶).
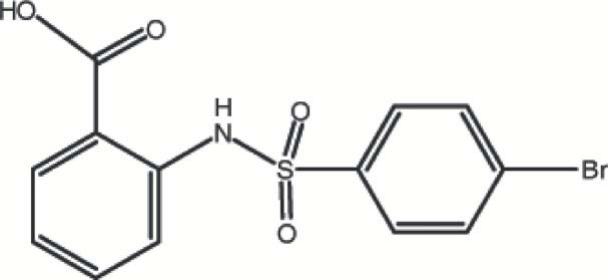


## Experimental

### 

#### Crystal data

C_13_H_10_BrNO_4_S
*M* *_r_* = 356.19
Monoclinic, 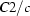

*a* = 27.8316 (11) Å
*b* = 8.5684 (4) Å
*c* = 11.6632 (5) Å
β = 98.196 (2)°
*V* = 2752.9 (2) Å^3^
*Z* = 8
Mo *K*α radiation
μ = 3.15 mm^−1^
*T* = 296 K
0.23 × 0.19 × 0.11 mm

#### Data collection

Bruker Kappa-APEXII CCD area-detector diffractometer
Absorption correction: refined from Δ*F* [Cubic fit to sin(θ)/λ - 24 parameters; Parkin *et al.*, 1995 ▶] *T*_min_ = 0.497, *T*_max_ = 0.707
14822 measured reflections
3416 independent reflections
1764 reflections with *I* > 2σ(*I*)
*R*_int_ = 0.042

#### Refinement

*R*[*F* ^2^ > 2σ(*F* ^2^)] = 0.038
*wR*(*F* ^2^) = 0.093
*S* = 0.98
3416 reflections
182 parameters
H-atom parameters constrained
Δρ_max_ = 0.27 e Å^−3^
Δρ_min_ = −0.29 e Å^−3^

### 

Data collection: *APEX2* (Bruker, 2007 ▶); cell refinement: *SAINT* (Bruker, 2007 ▶); data reduction: *SAINT*; program(s) used to solve structure: *SIR97* (Altomare *et al.*, 1999 ▶); program(s) used to refine structure: *SHELXL97* (Sheldrick, 2008 ▶); molecular graphics: *ORTEP-3 for Windows* (Farrugia, 1997 ▶); software used to prepare material for publication: *WinGX* (Farrugia, 1999 ▶) and *PLATON* (Spek, 2009 ▶).

## Supplementary Material

Crystal structure: contains datablocks global, I. DOI: 10.1107/S1600536809022545/is2431sup1.cif

Structure factors: contains datablocks I. DOI: 10.1107/S1600536809022545/is2431Isup2.hkl

Additional supplementary materials:  crystallographic information; 3D view; checkCIF report

## Figures and Tables

**Table 1:** Hydrogen-bond geometry (Å, °)

*D*—H⋯*A*	*D*—H	H⋯*A*	*D*⋯*A*	*D*—H⋯*A*
N1—H1⋯O1	0.86	2.13	2.670 (3)	121
O2—H2*A*⋯O1^i^	0.82	1.89	2.703 (3)	173
C6—H6⋯O4^ii^	0.93	2.56	3.185 (4)	125
C11—H11⋯O3^iii^	0.93	2.47	3.369 (3)	164
C12—H12⋯O4	0.93	2.31	2.987 (3)	130

Symmetry codes: (i) 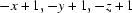; (ii) 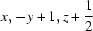; (iii) 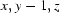.

**Fig. 1.**
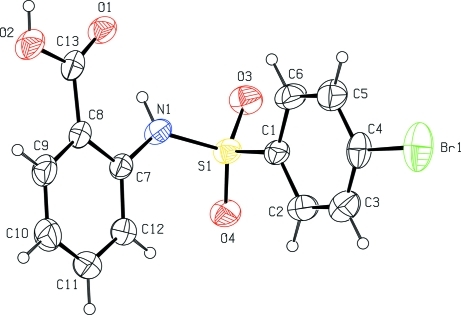
View of the title molecule showing the atom labelling scheme and displacement ellipsoids at the 30% probability level.

**Fig. 2.**
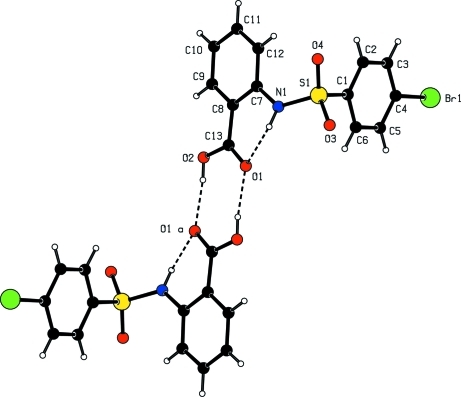
View of the dimeric structure of (I). Hydrogen bonds are indicated by dashed lines. The atom labelled with the suffix a is generated by the symmetry operator (3/2 - x,1/2 - y,1 - z).

**Fig. 3.**
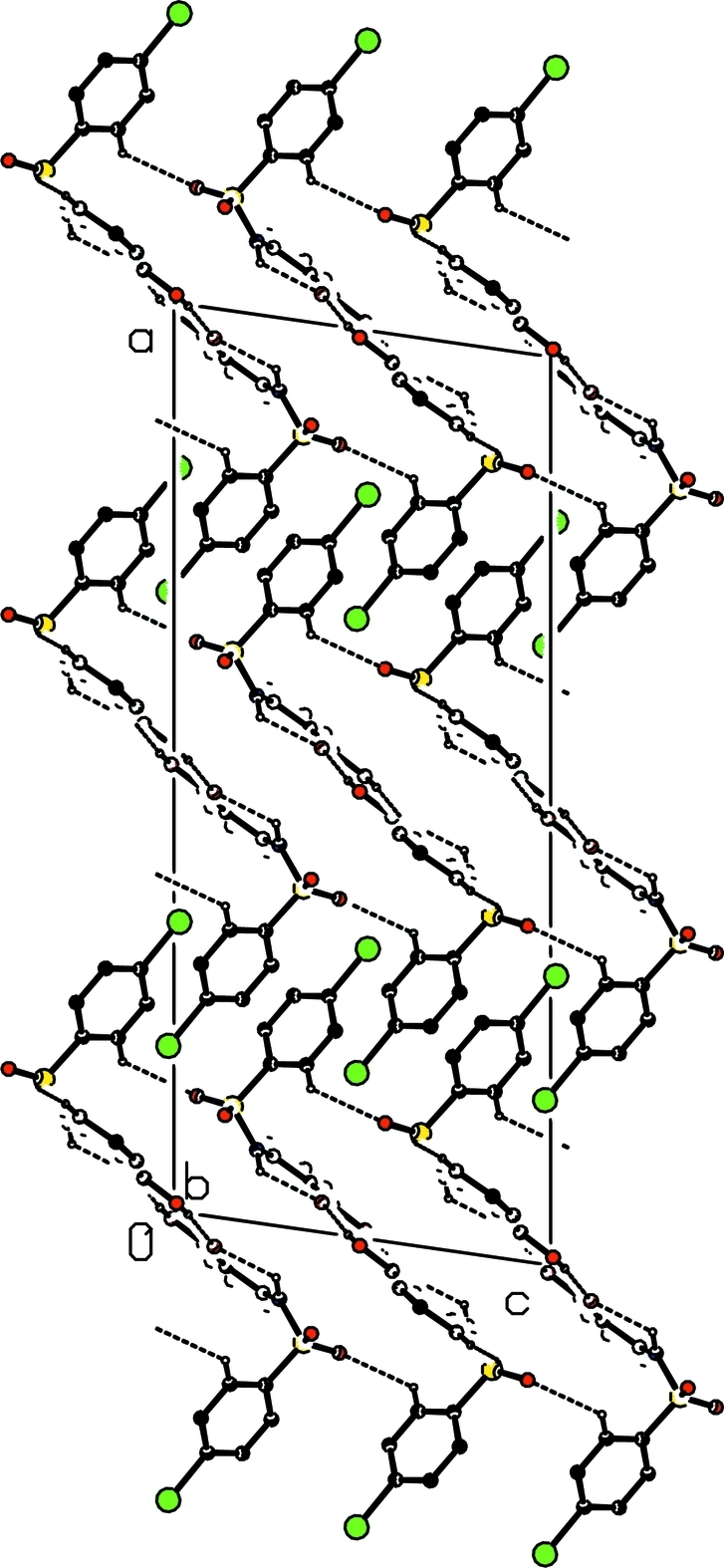
The molecular packing and hydrogen bonding for (I), viewed down the b axis. Hydrogen atoms not involved in the showed interactions have been omitted for clarity.

## supplementary crystallographic information

### Comment

Sulfonamides are biologically active organic compounds. The anthranilic sulfonamide derivative has been reported as inhibitors of Methionine aminopeptidase-2 (MetAP2) (Sheppard *et al.*, 2006) and halogenated anthranilic sulfonamide derivatives have been identified as novel, selective Cholecystokinin-2 Receptor Antagonists (Allison *et al.*, 2006). The title compound is halogenated sulfonamide in countinuation of our studies on the synthesis of sulfonamide derivatives (Arshad *et al.*, 2009) and benzothiazines (Shafiq *et al.*, 2009).

In the title compound, (I), (Fig. 1), the values of the geometric parameters are normal, and they are comparable with those in the reported structure of the isomorf compound 2-benzenesulfonamidobenzoic acid (Asiri *et al.*, 2009). The angle between the benzene rings is 82.75 (15)°.

The crystal packing is stabilized by C—H···O and O—H···O hydrogen bonds (Table 1). The intramolecular N—H···O hydrogen bond generates a graph set motif S(6). The O—H···O hydrogen bond forms a cyclic dimer, with a *R*^2^_2_(8) motif (Bernstein *et al.*, 1995; Etter, 1990), about a inversion centre (Fig. 2). Figure 3 shows the molecular packing for (I) viewed down the *b* axis, generating a zigzag layer running along the *a* axis.

### Experimental

Anthranilic acid (2 g, 14.6 mmol) was dissolved in distilled water (10 ml) in a round bottom flask (25 ml). The pH of the solution was maintained at 8–9 using 1*M*, Na_2_CO_3_. 4- Bromobenzene sulfonylchloride (3.72 g, 14.6 mmol) was suspended to the above solution and stirred at room temperature until all the 4-bromobenzene sulfonyl chloride was consumed. Progress of the reaction was observed by disappearing of suspension to clear solution. On completion of the reaction the pH was adjusted 1–2, using 1 N HCl. The precipitate obtained was filtered, washed with distilled water, dried and recrystallized in methanol to yield dark brown crystals.

### Refinement

H atoms were fixed geometrically and treated as riding, with C—H = 0.93 Å, N—H = 0.86 Å and O—H = 0.82 Å, and with *U*_iso_(H) = 1.2*U*_eq_(C, N) and *U*_iso_(H) = 1.5*U*_eq_(O).

### Crystal data

**Table:**

C_13_H_10_BrNO_4_S	*F*(000) = 1424
*M**_r_* = 356.19	*D*_x_ = 1.719 Mg m^−^^3^
Monoclinic, *C*2/*c*	Mo *K*α radiation, λ = 0.71073 Å
Hall symbol: -C 2yc	Cell parameters from 3121 reflections
*a* = 27.8316 (11) Å	θ = 2.5–22.0°
*b* = 8.5684 (4) Å	µ = 3.15 mm^−^^1^
*c* = 11.6632 (5) Å	*T* = 296 K
β = 98.196 (2)°	Block, dark brown
*V* = 2752.9 (2) Å^3^	0.23 × 0.19 × 0.11 mm
*Z* = 8	

### Data collection

**Table:**

Bruker Kappa-APEXII CCD area-detector diffractometer	3416 independent reflections
Radiation source: sealed tube	1764 reflections with *I* > 2σ(*I*)
graphite	*R*_int_ = 0.042
φ and ω scans	θ_max_ = 28.3°, θ_min_ = 2.5°
Absorption correction: part of the refinement model (Δ*F*) [Cubic fit to sin(θ)/λ - 24 parameters; Parkin *et al.*, 1995]	*h* = −37→36
*T*_min_ = 0.497, *T*_max_ = 0.707	*k* = −11→11
14822 measured reflections	*l* = −15→15

### Refinement

**Table:**

Refinement on *F*^2^	Primary atom site location: structure-invariant direct methods
Least-squares matrix: full	Secondary atom site location: difference Fourier map
*R*[*F*^2^ > 2σ(*F*^2^)] = 0.038	Hydrogen site location: inferred from neighbouring sites
*wR*(*F*^2^) = 0.093	H-atom parameters constrained
*S* = 0.98	*w* = 1/[σ^2^(*F*_o_^2^) + (0.033*P*)^2^ + 1.5317*P*] where *P* = (*F*_o_^2^ + 2*F*_c_^2^)/3
3416 reflections	(Δ/σ)_max_ = 0.001
182 parameters	Δρ_max_ = 0.27 e Å^−^^3^
0 restraints	Δρ_min_ = −0.29 e Å^−^^3^

### Special details

**Table:**

Geometry. Bond distances, angles *etc*. have been calculated using the rounded fractional coordinates. All su's are estimated from the variances of the (full) variance-covariance matrix. The cell e.s.d.'s are taken into account in the estimation of distances, angles and torsion angles
Refinement. Refinement on *F*^2^ for ALL reflections except those flagged by the user for potential systematic errors. Weighted *R*-factors *wR* and all goodnesses of fit *S* are based on *F*^2^, conventional *R*-factors *R* are based on *F*, with *F* set to zero for negative *F*^2^. The observed criterion of *F*^2^ > σ(*F*^2^) is used only for calculating -*R*-factor-obs *etc*. and is not relevant to the choice of reflections for refinement. *R*-factors based on *F*^2^ are statistically about twice as large as those based on *F*, and *R*-factors based on ALL data will be even larger.

### Fractional atomic coordinates and isotropic or equivalent isotropic displacement parameters (Å^2^)

**Table:**

	*x*	*y*	*z*	*U*_iso_*/*U*_eq_	
Br1	0.81917 (1)	0.43825 (6)	0.51473 (4)	0.1153 (2)	
S1	0.62425 (3)	0.44674 (8)	0.15544 (6)	0.0547 (2)	
O1	0.53281 (7)	0.4722 (2)	0.39261 (16)	0.0628 (7)	
O2	0.50891 (7)	0.2889 (2)	0.50674 (17)	0.0721 (8)	
O3	0.61345 (8)	0.6071 (2)	0.13563 (17)	0.0713 (8)	
O4	0.63014 (7)	0.3473 (2)	0.06120 (15)	0.0665 (7)	
N1	0.58022 (7)	0.3824 (2)	0.22029 (18)	0.0555 (8)	
C1	0.67716 (9)	0.4336 (3)	0.2571 (2)	0.0514 (9)	
C2	0.71403 (11)	0.3319 (3)	0.2413 (3)	0.0659 (11)	
C3	0.75605 (11)	0.3324 (4)	0.3185 (3)	0.0788 (14)	
C4	0.76082 (11)	0.4310 (4)	0.4111 (3)	0.0705 (11)	
C5	0.72375 (12)	0.5304 (4)	0.4281 (3)	0.0782 (14)	
C6	0.68182 (12)	0.5303 (4)	0.3517 (3)	0.0714 (12)	
C7	0.57612 (8)	0.2291 (3)	0.2636 (2)	0.0488 (9)	
C8	0.55269 (8)	0.2054 (3)	0.3611 (2)	0.0505 (9)	
C9	0.54903 (10)	0.0538 (4)	0.4003 (3)	0.0685 (11)	
C10	0.56676 (11)	−0.0710 (4)	0.3463 (3)	0.0788 (14)	
C11	0.58873 (11)	−0.0459 (3)	0.2499 (3)	0.0726 (11)	
C12	0.59331 (10)	0.1022 (3)	0.2086 (3)	0.0606 (10)	
C13	0.53137 (9)	0.3347 (4)	0.4206 (2)	0.0562 (10)	
H1	0.55740	0.44700	0.22910	0.0670*	
H2	0.71040	0.26350	0.17880	0.0790*	
H2A	0.49850	0.36530	0.53760	0.1080*	
H3	0.78130	0.26550	0.30760	0.0940*	
H5	0.72720	0.59740	0.49150	0.0940*	
H6	0.65640	0.59580	0.36380	0.0860*	
H9	0.53410	0.03640	0.46550	0.0820*	
H10	0.56390	−0.17150	0.37460	0.0940*	
H11	0.60060	−0.13020	0.21220	0.0870*	
H12	0.60810	0.11760	0.14300	0.0730*	

### Atomic displacement parameters (Å^2^)

**Table:**

	*U*^11^	*U*^22^	*U*^33^	*U*^12^	*U*^13^	*U*^23^
Br1	0.0724 (2)	0.1642 (5)	0.1042 (4)	−0.0277 (2)	−0.0048 (2)	0.0332 (3)
S1	0.0662 (4)	0.0525 (4)	0.0493 (4)	0.0040 (3)	0.0221 (3)	0.0044 (3)
O1	0.0667 (12)	0.0678 (13)	0.0586 (12)	0.0035 (10)	0.0251 (10)	0.0005 (10)
O2	0.0768 (13)	0.0831 (13)	0.0641 (13)	−0.0003 (11)	0.0362 (11)	0.0049 (11)
O3	0.0920 (14)	0.0549 (12)	0.0700 (14)	0.0115 (10)	0.0218 (11)	0.0149 (10)
O4	0.0860 (13)	0.0710 (12)	0.0478 (11)	−0.0033 (10)	0.0273 (10)	−0.0051 (10)
N1	0.0548 (12)	0.0553 (13)	0.0600 (15)	0.0105 (10)	0.0206 (11)	0.0067 (11)
C1	0.0595 (15)	0.0492 (15)	0.0499 (16)	−0.0006 (12)	0.0226 (13)	0.0021 (13)
C2	0.0743 (19)	0.0599 (18)	0.066 (2)	0.0077 (15)	0.0182 (16)	−0.0043 (15)
C3	0.069 (2)	0.084 (2)	0.086 (3)	0.0129 (17)	0.0198 (19)	0.006 (2)
C4	0.0598 (17)	0.089 (2)	0.064 (2)	−0.0170 (17)	0.0137 (15)	0.0162 (18)
C5	0.076 (2)	0.100 (3)	0.061 (2)	−0.0116 (19)	0.0177 (18)	−0.0176 (18)
C6	0.074 (2)	0.079 (2)	0.065 (2)	0.0075 (16)	0.0227 (17)	−0.0166 (17)
C7	0.0403 (13)	0.0554 (16)	0.0510 (16)	−0.0008 (11)	0.0079 (12)	−0.0010 (13)
C8	0.0388 (12)	0.0612 (17)	0.0529 (16)	−0.0032 (12)	0.0112 (11)	0.0012 (14)
C9	0.0602 (17)	0.073 (2)	0.077 (2)	−0.0094 (15)	0.0259 (16)	0.0083 (18)
C10	0.074 (2)	0.0595 (19)	0.106 (3)	−0.0088 (16)	0.024 (2)	0.0123 (19)
C11	0.076 (2)	0.0557 (19)	0.090 (2)	−0.0043 (15)	0.0251 (18)	−0.0065 (17)
C12	0.0607 (17)	0.0576 (18)	0.0665 (19)	−0.0056 (13)	0.0196 (14)	−0.0032 (15)
C13	0.0402 (13)	0.080 (2)	0.0490 (16)	−0.0046 (13)	0.0080 (12)	0.0015 (15)

### Geometric parameters (Å, °)

**Table:**

Br1—C4	1.882 (3)	C7—C12	1.382 (4)
S1—O3	1.4184 (19)	C7—C8	1.404 (3)
S1—O4	1.4186 (19)	C8—C13	1.476 (4)
S1—N1	1.625 (2)	C8—C9	1.386 (4)
S1—C1	1.758 (3)	C9—C10	1.368 (5)
O1—C13	1.225 (4)	C10—C11	1.371 (5)
O2—C13	1.317 (3)	C11—C12	1.370 (4)
O2—H2A	0.8200	C2—H2	0.9300
N1—C7	1.418 (3)	C3—H3	0.9300
N1—H1	0.8600	C5—H5	0.9300
C1—C6	1.371 (4)	C6—H6	0.9300
C1—C2	1.379 (4)	C9—H9	0.9300
C2—C3	1.371 (5)	C10—H10	0.9300
C3—C4	1.363 (5)	C11—H11	0.9300
C4—C5	1.374 (5)	C12—H12	0.9300
C5—C6	1.364 (5)		
			
Br1···C9^i^	3.662 (3)	C12···O4	2.987 (3)
Br1···C10^i^	3.540 (3)	C13···N1^vi^	3.325 (3)
Br1···C11^i^	3.599 (3)	C13···O3^iii^	3.182 (3)
S1···H12	2.8600	C13···C7^vi^	3.543 (3)
O1···N1	2.670 (3)	C2···H5^v^	3.0500
O1···O2^ii^	2.703 (3)	C5···H2^ix^	3.0900
O2···O3^iii^	3.205 (3)	C6···H3^ix^	3.0200
O2···O1^ii^	2.703 (3)	C9···H9^vii^	3.0700
O3···C11^iv^	3.369 (3)	C13···H1	2.6300
O3···C13^v^	3.182 (3)	C13···H1^vi^	2.9700
O3···O2^v^	3.205 (3)	C13···H2A^ii^	2.7700
O4···C6^v^	3.185 (4)	H1···O1	2.1300
O4···C5^v^	3.384 (4)	H1···C13	2.6300
O4···C12	2.987 (3)	H1···O1^vi^	2.7100
O1···H1	2.1300	H1···C13^vi^	2.9700
O1···H2A^ii^	1.8900	H2···O4	2.5500
O1···H1^vi^	2.7100	H2···H5^v^	2.5900
O2···H9	2.3500	H2···C5^x^	3.0900
O2···H10^vii^	2.8000	H2A···O1^ii^	1.8900
O3···H11^iv^	2.4700	H2A···C13^ii^	2.7700
O3···H6	2.7600	H2A···H2A^ii^	2.4700
O4···H12	2.3100	H3···C6^x^	3.0200
O4···H2	2.5500	H5···C2^iii^	3.0500
O4···H6^v^	2.5600	H5···H2^iii^	2.5900
N1···O1	2.670 (3)	H6···O3	2.7600
N1···C13^vi^	3.325 (3)	H6···O4^iii^	2.5600
C5···O4^iii^	3.384 (4)	H9···O2	2.3500
C6···O4^iii^	3.185 (4)	H9···C9^vii^	3.0700
C7···C13^vi^	3.543 (3)	H9···H9^vii^	2.2500
C9···Br1^i^	3.661 (3)	H10···O2^vii^	2.8000
C10···Br1^i^	3.540 (3)	H11···O3^viii^	2.4700
C11···Br1^i^	3.599 (3)	H12···S1	2.8600
C11···O3^viii^	3.369 (3)	H12···O4	2.3100
			
O3—S1—O4	120.06 (12)	C9—C8—C13	119.7 (2)
O3—S1—N1	104.36 (12)	C8—C9—C10	122.2 (3)
O3—S1—C1	108.04 (13)	C9—C10—C11	119.2 (3)
O4—S1—N1	109.57 (11)	C10—C11—C12	120.6 (3)
O4—S1—C1	107.82 (12)	C7—C12—C11	120.7 (3)
N1—S1—C1	106.21 (11)	O1—C13—O2	121.8 (3)
C13—O2—H2A	109.00	O1—C13—C8	124.5 (2)
S1—N1—C7	125.76 (16)	O2—C13—C8	113.7 (3)
C7—N1—H1	117.00	C1—C2—H2	120.00
S1—N1—H1	117.00	C3—C2—H2	120.00
S1—C1—C2	121.2 (2)	C2—C3—H3	120.00
S1—C1—C6	118.6 (2)	C4—C3—H3	120.00
C2—C1—C6	120.2 (3)	C4—C5—H5	120.00
C1—C2—C3	119.5 (3)	C6—C5—H5	120.00
C2—C3—C4	120.0 (3)	C1—C6—H6	120.00
Br1—C4—C3	120.5 (2)	C5—C6—H6	120.00
Br1—C4—C5	118.9 (3)	C8—C9—H9	119.00
C3—C4—C5	120.6 (3)	C10—C9—H9	119.00
C4—C5—C6	119.7 (3)	C9—C10—H10	120.00
C1—C6—C5	120.0 (3)	C11—C10—H10	120.00
C8—C7—C12	119.6 (2)	C10—C11—H11	120.00
N1—C7—C12	120.9 (2)	C12—C11—H11	120.00
N1—C7—C8	119.5 (2)	C7—C12—H12	120.00
C7—C8—C9	117.9 (2)	C11—C12—H12	120.00
C7—C8—C13	122.5 (2)		
			
O3—S1—N1—C7	−176.5 (2)	Br1—C4—C5—C6	177.9 (3)
O4—S1—N1—C7	53.8 (2)	C3—C4—C5—C6	0.0 (5)
C1—S1—N1—C7	−62.4 (2)	C4—C5—C6—C1	−1.3 (5)
O3—S1—C1—C2	−135.0 (2)	N1—C7—C8—C9	179.5 (2)
O4—S1—C1—C2	−3.8 (3)	N1—C7—C8—C13	0.7 (3)
N1—S1—C1—C2	113.6 (2)	C12—C7—C8—C9	1.8 (4)
O3—S1—C1—C6	43.2 (3)	C12—C7—C8—C13	−177.0 (2)
O4—S1—C1—C6	174.3 (2)	N1—C7—C12—C11	−179.2 (3)
N1—S1—C1—C6	−68.3 (3)	C8—C7—C12—C11	−1.6 (4)
S1—N1—C7—C8	149.12 (19)	C7—C8—C9—C10	−0.9 (4)
S1—N1—C7—C12	−33.3 (3)	C13—C8—C9—C10	177.9 (3)
S1—C1—C6—C5	−175.6 (3)	C7—C8—C13—O1	−1.4 (4)
S1—C1—C2—C3	175.7 (2)	C7—C8—C13—O2	176.9 (2)
C6—C1—C2—C3	−2.4 (4)	C9—C8—C13—O1	179.9 (3)
C2—C1—C6—C5	2.5 (5)	C9—C8—C13—O2	−1.9 (3)
C1—C2—C3—C4	1.1 (5)	C8—C9—C10—C11	−0.3 (5)
C2—C3—C4—C5	0.1 (5)	C9—C10—C11—C12	0.6 (5)
C2—C3—C4—Br1	−177.7 (2)	C10—C11—C12—C7	0.3 (5)

Symmetry codes: (i) −*x*+3/2, −*y*+1/2, −*z*+1; (ii) −*x*+1, −*y*+1, −*z*+1; (iii) *x*, −*y*+1, *z*+1/2; (iv) *x*, *y*+1, *z*; (v) *x*, −*y*+1, *z*−1/2; (vi) −*x*+1, *y*, −*z*+1/2; (vii) −*x*+1, −*y*, −*z*+1; (viii) *x*, *y*−1, *z*; (ix) −*x*+3/2, *y*+1/2, −*z*+1/2; (x) −*x*+3/2, *y*−1/2, −*z*+1/2.

### Hydrogen-bond geometry (Å, °)

**Table:**

*D*—H···*A*	*D*—H	H···*A*	*D*···*A*	*D*—H···*A*
N1—H1···O1	0.86	2.13	2.670 (3)	121
O2—H2A···O1^ii^	0.82	1.89	2.703 (3)	173
C6—H6···O4^iii^	0.93	2.56	3.185 (4)	125
C11—H11···O3^viii^	0.93	2.47	3.369 (3)	164
C12—H12···O4	0.93	2.31	2.987 (3)	130

Symmetry codes: (ii) −*x*+1, −*y*+1, −*z*+1; (iii) *x*, −*y*+1, *z*+1/2; (viii) *x*, *y*−1, *z*.
